# A novel technique for computer-assisted dental autotransplantation: A case report with 42 months of follow-up

**DOI:** 10.34172/japid.025.3760

**Published:** 2025-04-13

**Authors:** Ehsan Chegeni, José Marques, Rui Figueiredo, Eduard Valmaseda-Castellón, Mahdi Kadkhodazadeh

**Affiliations:** ^1^Faculty of Medicine and Health Sciences, University of Barcelona, Barcelona, Spain; ^2^Department of Periodontics, School of Dentistry, Shahid Beheshti University of Medical Sciences, Tehran, Iran; ^3^Dental and Maxillofacial Pathology and Therapeutics Research Group, IDIBELL Research Institute, Barcelona, Spain; ^4^Research Institute of Dental Sciences, School of Dentistry, Shahid Beheshti University of Medical Sciences, Tehran, Iran

**Keywords:** Autologous transplantation, Case report, Dental implant, Third molar

## Abstract

Dental autotransplantation (DAT) is an effective technique for replacing missing teeth. This case report presents a novel computer-assisted approach using cone-beam computed tomography (CBCT) for 3D planning. A 15-year-old female with a hopeless molar due to failed endodontic treatment underwent DAT. Following 3D planning, the donor tooth was surgically extracted and transplanted into the prepared socket, followed by semi-rigid splinting. A meticulous 42-month clinical and radiographic follow-up demonstrated the success and stability of the procedure. This innovative method emphasizes the role of advanced computer technology and 3D imaging in enhancing surgical precision and treatment outcomes. Within the limitations of this case report, DAT, combined with computer-assisted planning, proved a reliable and predictable treatment option for replacing hopeless teeth, particularly in young patients. This approach showed the potential of DAT to transform tooth replacement strategies with better accuracy and decision-making.

## Introduction

 Dental autotransplantation (DAT) involves placing a recently extracted tooth in another location to replace missing or severely damaged teeth within the same individual. Originating in the mid-20th century, the concept of DAT began with early attempts to move teeth within the same individual.^1,2^With advancements in surgical techniques and a deeper understanding of tooth development, DAT has evolved from a pioneering concept to a recognized and viable approach in contemporary dentistry. The success of DAT depends on several factors: careful case selection, a precise surgical technique, an adequate root development stage, correct bone management, and endodontic treatment when necessary.^3-5^

 Studies emphasize the importance of patient age since younger individuals in their early to mid-teenage years seem to experience more favorable outcomes.^6^ Success rates are further enhanced by selecting donor teeth with similar root morphology and size.^7^ For over fifty years, third molars have been successfully transplanted, offering a viable option for replacing first or second molars with a reasonable prognosis.^7,8^ The long-term survival of transplanted teeth is contingent upon successful integration into their new environment and the formation of new bone around the root.^9-11^

 DAT is applicable in various clinical scenarios, offering a versatile option for tooth replacement.^7^ Common indications include congenitally missing teeth, traumatic injuries resulting in tooth loss, and cases where traditional prosthetic options are not feasible or desirable.^8^ The procedure is particularly advantageous in young patients with developing dentition, providing a natural and functional replacement for missing or compromised teeth.^8^ Moreover, DAT becomes a viable alternative when conventional treatments such as dental implants or fixed prosthetics are not suitable due to anatomical constraints, patient preferences, or financial considerations.^8^

 While DAT presents a promising solution, careful consideration of contraindications is crucial. Patients with systemic conditions affecting healing, such as uncontrolled diabetes or immunodeficiency disorders, may not be ideal candidates.^12^ Individuals with severe periodontal disease, inadequate bone support, or compromised oral hygiene may face increased risks and reduced success rates. Thorough patient assessment is essential to identify and address contraindications, ensuring optimal outcomes. Postoperative care precautions include close monitoring, guidance on oral hygiene practices, and instructions to avoid excessive force on the transplanted tooth during the initial healing phase.^13,14^

 As dentistry evolves, DAT remains a dynamic and patient-oriented solution for addressing various dental conditions, contributing to aesthetics and long-term oral health.

 Over the years, multiple approaches have been developed, from traditional methods relying on two-dimensional radiographs to modern technologies becoming more affordable and precise.^15^The present case report aims to illustrate the feasibility of DAT surgery using a single DICOM image from the patient.

## Case Report

 A 15-year-old female was referred to the Department of Oral Surgery and Orofacial Implantology at the Dental Hospital of the University of Barcelona, presenting pain in the lower right molar. The patient reported that the tooth had undergone an initial endodontic treatment just before the Covid-19 pandemic lockdown. The patient did not refer to any systemic disorders or the use of medications.

**Figure 1:**
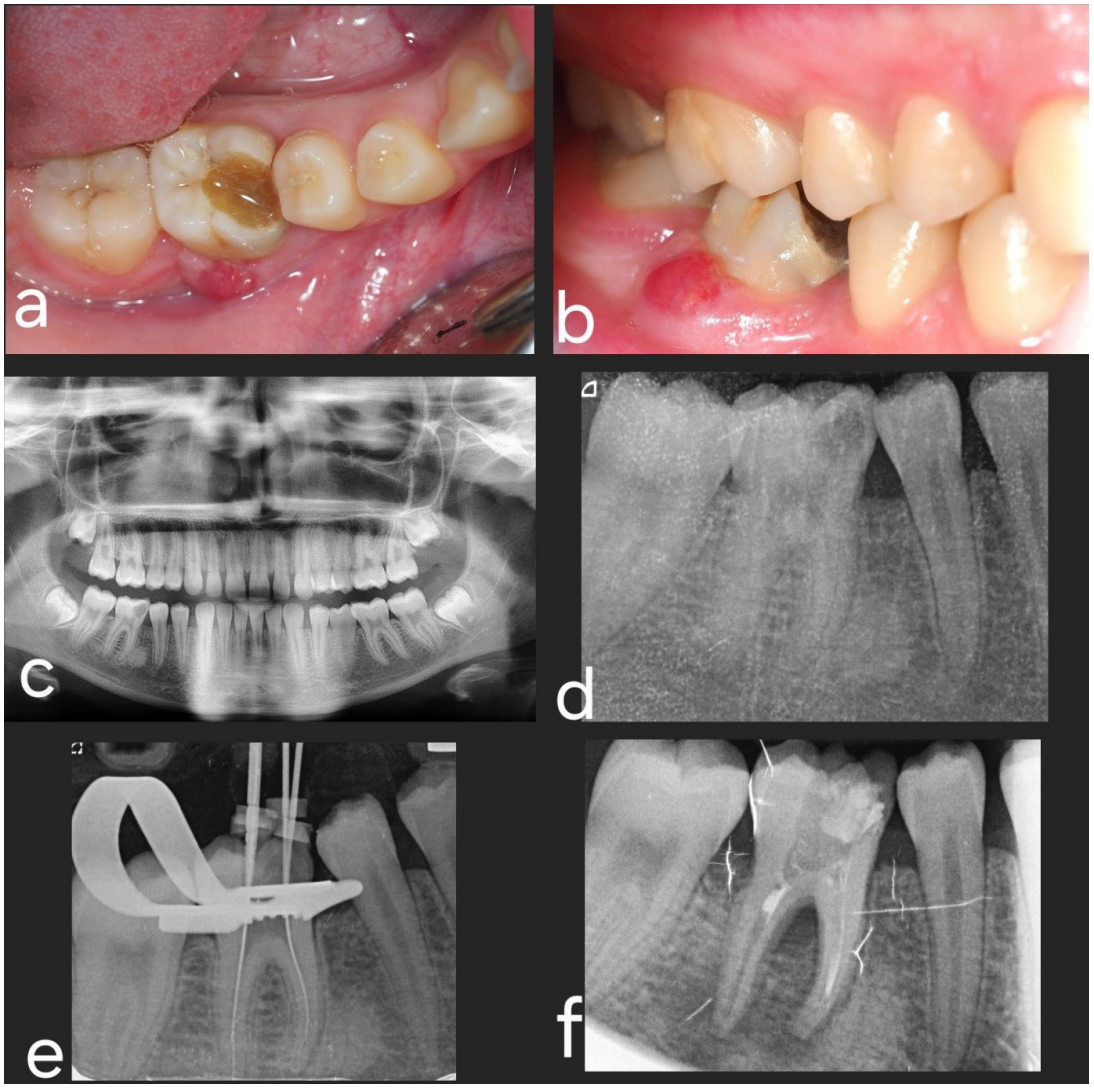
Diagnosis and planning. (a and b) Preoperative intraoral pictures; occlusal and lateral views. (c) Panoramic view before endodontic treatment. (d and e) Periapical radiographs during endodontic treatment. (f) New periapical radiograph before autotransplantation

 Upon intraoral examination, inflammation was observed on the buccal side of tooth #4.6 (lower right first molar), accompanied by pain upon pressure and the presence of purulent drainage. Bleeding on probing was also registered, and a pocket depth of 6 mm on the buccal side of the affected tooth was observed (Figure 1). The Endodontics Department established a provisional diagnosis of vertical root fracture with furcal involvement and infection, recommending dental extraction as the preferred treatment. The DAT option was introduced after thorough discussions with the patient and her parents. This alternative was considered since both teeth (left lower third molar and right lower first molar) needed extraction. Considering the patient’s age, dental implant placement was deemed contraindicated. A cone-beam computed tomographic (CBCT) scan was subsequently requested to evaluate the case further.

**Figure 2:**
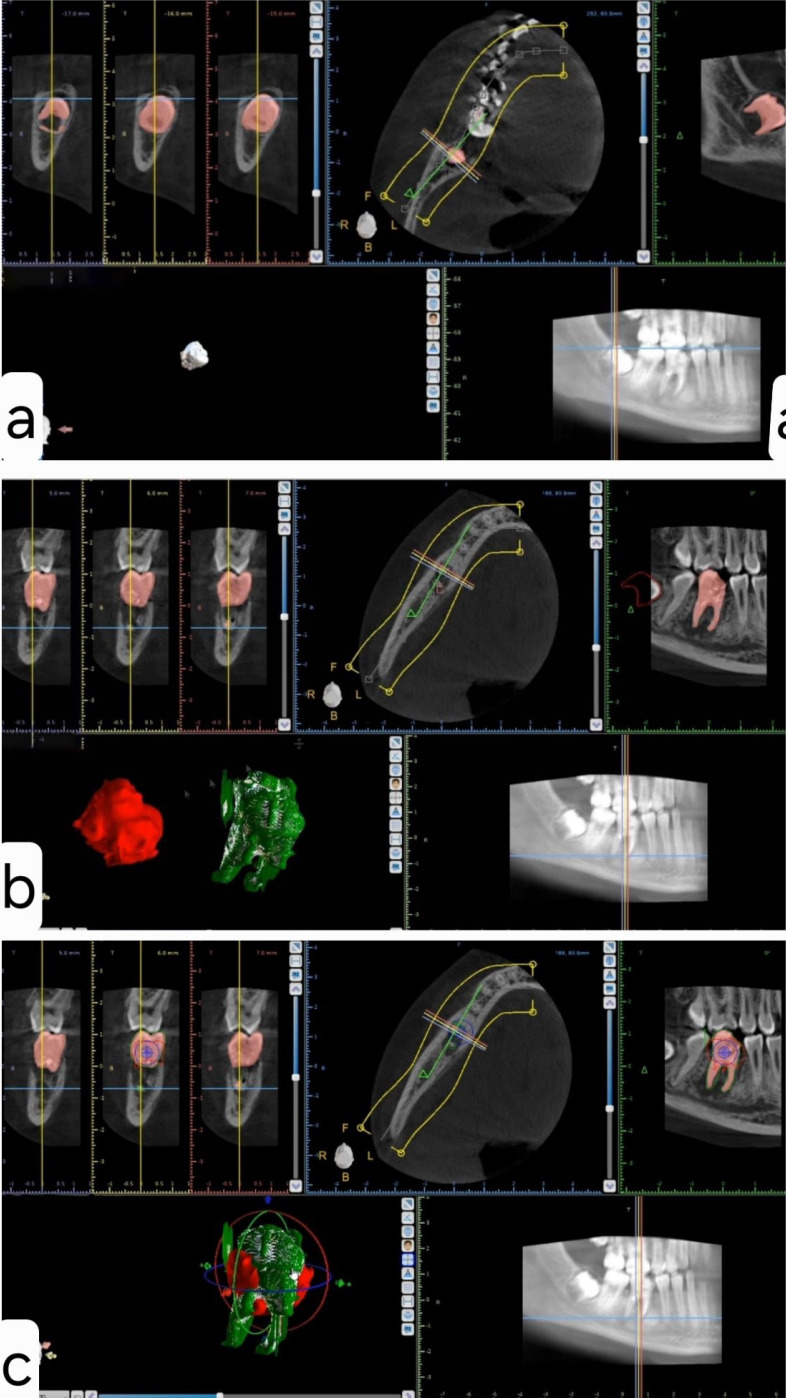
Preoperative digital analysis. (a) Isolation of the third molar (donor tooth) and generation of a three-dimensional (3D) model. The pink color represents the donor tooth marked for 3D modeling. (b) Isolation of the receptor tooth and generation of a 3D model. The receptor tooth is represented in green, and the donor tooth in red. (c) Merging and positioning the donor tooth (red) into the receptor zone (green) to assess feasibility and placement post-extraction

 The Digital Imaging and Communication in Medicine (DICOM) file was imported into the Blue Sky Plan software (BlueSkyBio, USA). The analytical process consisted of four sequential steps (Figure 2):

 Isolation of the donor tooth (third molar) and generation of a 3D model (Figure 2a): The initial step involved isolating the third molar (donor tooth) and generating a three-dimensional (3D) model to accurately represent its anatomical structure.

- Isolation of the receptor tooth (tooth #4.6) and creation of a 3D model (Figure 2b): Following the
- isolation of the donor tooth, a parallel process was undertaken for the receptor tooth (tooth #4.6) to create a corresponding 3D model.
- Placement of the donor tooth in the receptor zone and 3D measurements to assess feasibility (Figure 2c): This step involved placing the donor tooth in the receptor zone and conducting precise 3D measurements to assess the feasibility of the surgery. This step is critical for determining the optimal alignment and positioning of the transplanted tooth.
- Optional step: Exporting the data as an STL file to allow the fabrication of a 3D model impression of the donor tooth and prepare the receptor site according to the size and shape of the donor tooth before extracting the latter.
- Following a comprehensive analysis, a decision was made to use the lower left third molar as the first option to replace the hopeless first molar. In the event of complications, the right lower third molar, which also required extraction, could be used. The primary rationale behind this decision was that the left lower third molar had a more adequate root development stage.

The following steps were undertaken upon obtaining informed consent from the patient and her parents and administering 2 g of oral amoxicillin one hour before the intervention.

###  Preoperative antiseptic measures

- The patient performed a one-minute antiseptic mouthrinse using 0.12% chlorhexidine (Perioaid 0.12%; Dentaid, Cerdanyola del Vallès, Spain).
- Extraoral antisepsis involved applying a wet piece of gauze with 0.12% chlorhexidine.

###  Local anesthesia

 Right inferior alveolar nerve block and supplemental buccal infiltration using a 4% articaine solution with 1:100 000 epinephrine (Artinibsa; Inibsa, Lliçà de Vall, Spain).

### Extraction of the lower right first molar (receptor site)

**Figure 3:**
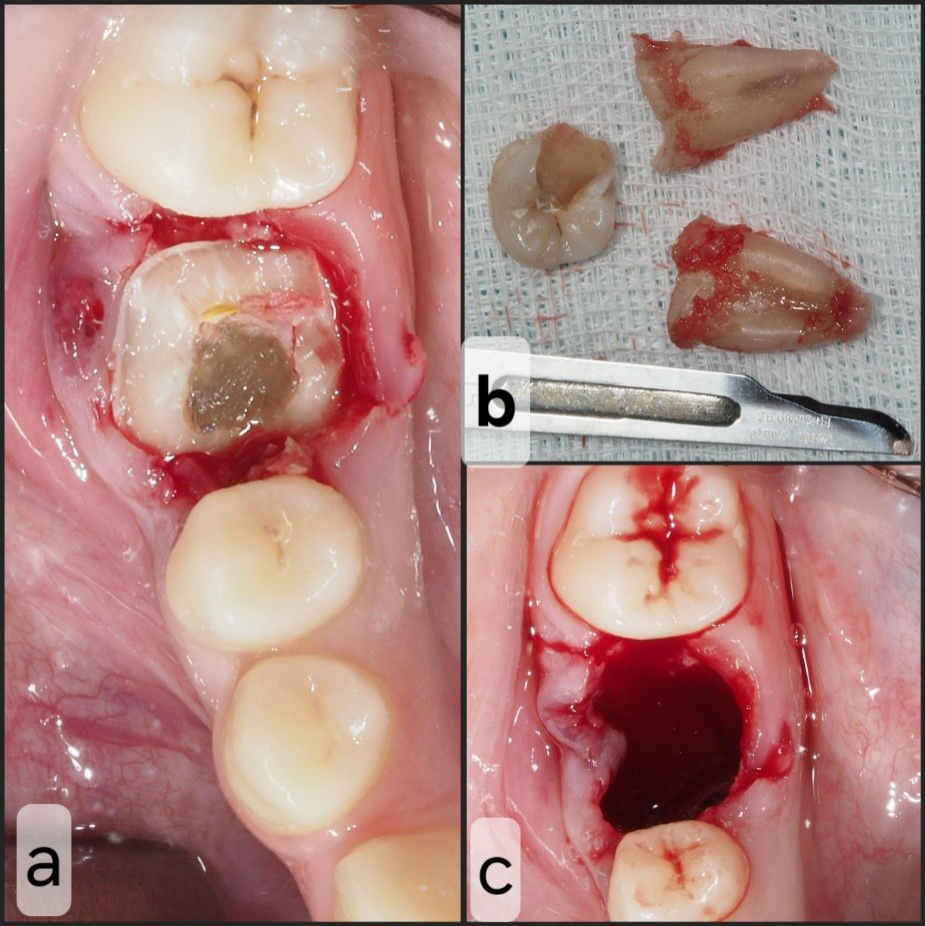
Receptor site procedure. (a) Receptor tooth after horizontal crown section. (b) Extracted tooth. (c) Receptor zone after extraction

A flapless horizontal crown sectioning under saline irrigation was performed using a 1:1 straight handpiece equipped with a #6 round tungsten bur, operating at 40 000 rpm. Subsequently, root sectioning was executed, and each root was individually extracted using straight small elevators. A careful curettage of the socket was carried out, followed by irrigation with a saline solution (Figure 3).

 Local mandibular block anesthesia on the left side was successfully achieved using 4% articaine with 1:100 000 epinephrine (Artinibsa; Inibsa, Lliçà de Vall, Spain). The surgical procedure was performed as follows.

###  Extraction of the lower left third molar (donor tooth)

 A full-thickness trapezoidal flap was raised, and buccal osteotomy was performed using a #6 round tungsten bur, operating at 40 000 rpm under saline solution irrigation. Afterward, the tooth was delicately removed using elevators, ensuring minimal contact with the roots to preserve its integrity. Curettage and thorough socket cleaning were performed to eliminate residual debris, and a 4-0 polyamide suture was used to close the wound.

###  Autotransplantation

**Figure 4:**
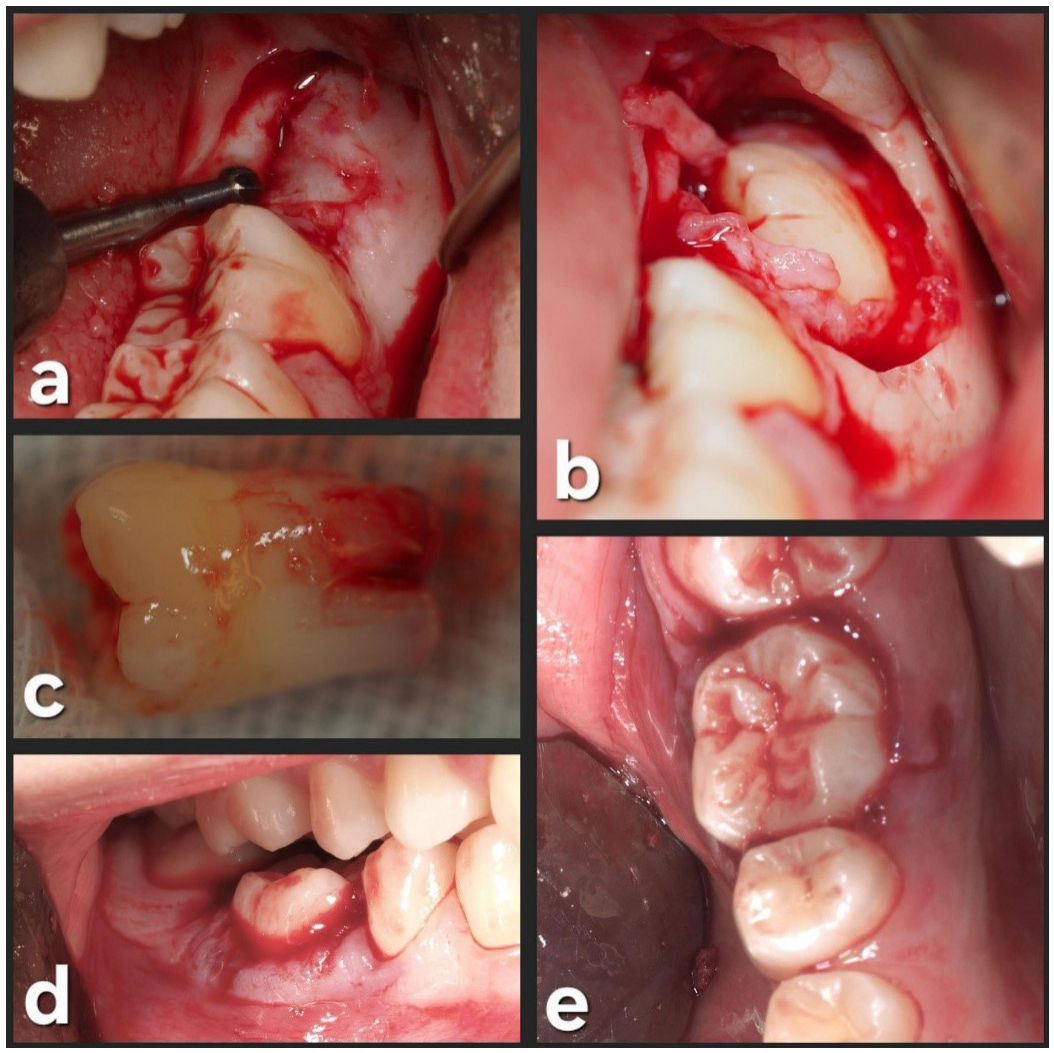
Donor site procedure. (a) Donor tooth zone. (b) Donor tooth exposure. (c) Extracted donor tooth. (d) Placement of the donor tooth into the receptor zone without occlusal interference. (e) Occlusal view of the receptor zone after autotransplantation

 Tooth #3.8 was carefully placed in the #4.6 socket in infraocclusion, and no socket modifications were required (Figure 4).

 This surgical approach, characterized by precise osteotomy, minimal trauma during tooth extraction, and meticulous socket management, allowed a successful autotransplantation. The preoperative computer-assisted analysis and the evaluation of the patient’s unique dental anatomy allowed the transplanted tooth to be placed with the need to modify the receptor site.

**Figure 5:**
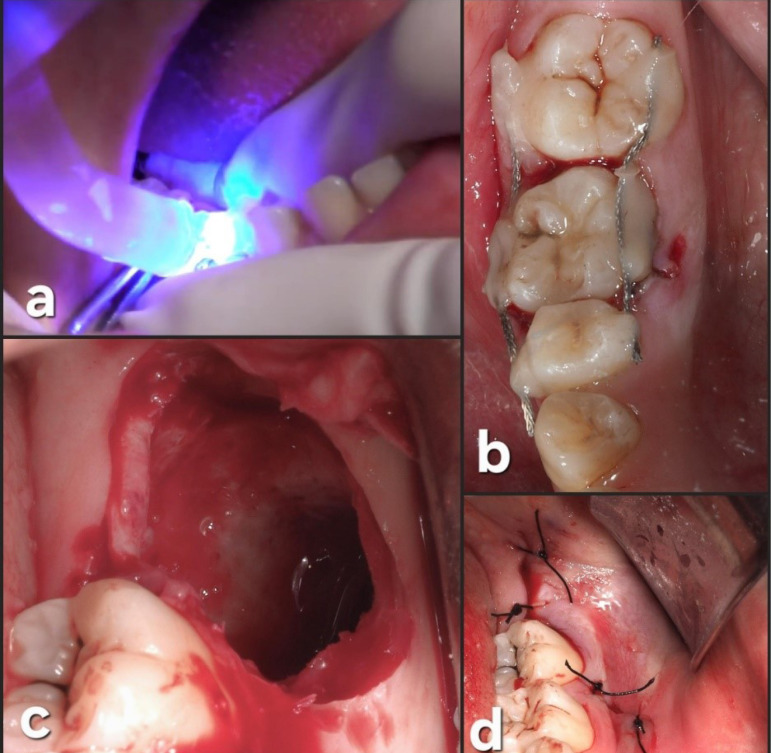
Final steps of the procedure. (a) Light-curing of the composites. (b) Occlusal view after semi-rigid splinting. (c) Donor site after extraction. (d) Donor site after suturing with 4.0 polyamide

 Afterward, a semi-rigid splinting of the transplanted and adjacent teeth (#4.5, #4.6, and #4.7) was made on the buccal and lingual sides. Etching was carried out using a 37% phosphoric acid gel (Ultra-Etch; Ultradent Products Inc., South Jordan, UT, USA) and bonding with OptiBond^TM^ (Kerr Dental, Orange, CA, USA), the composite resin (Tetric EvoFlow®, Ivoclar Vivodent S.L.U., Madrid, Spain) was placed on mid-buccal and mid-lingual areas of teeth and light-cured with Elipar^TM^ S10 LED light-curing device (3 M ESPE, Seefeld, Germany) for 30 seconds on each site. The donor site (left lower third molar area) was sutured with 4.0 polyamide suture (Supramid; B. Braun Melsungen AG, Melsungen, Germany) (Figure 5).

 Postoperative instructions were given to the patient, which included recommendations to follow a soft diet for 2 weeks, using ice packs intermittently for the initial 24 hours, and taking oral ibuprofen (600 mg) three times a day for 3‒5 days. As a rescue medication, paracetamol (1 g) was prescribed. The patient was also recommended to perform mouthrinses with 0.12% chlorhexidine twice daily for 14 days.

**Figure 6:**
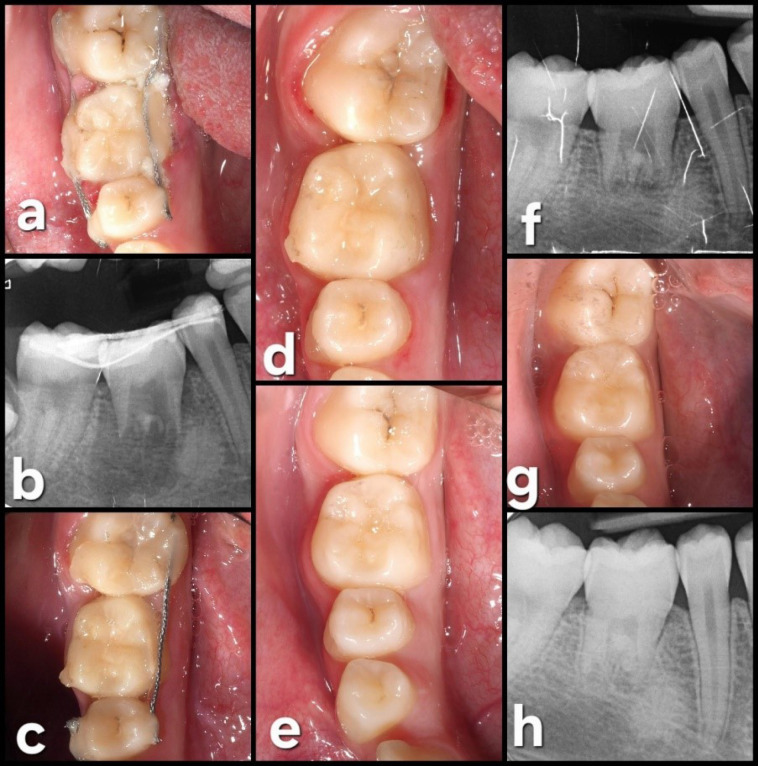
Follow-up. (a) One week after surgery. (b) Periapical radiograph one week after surgery. (c) Four weeks after surgery, the buccal splinting has fallen off. (d) Four weeks after surgery, the splinting was removed. (e) Six months after surgery. (f) Periapical radiograph six months after surgery. (g) Fourteen-month follow-up. (h) Periapical radiograph 14 months after surgery

 The initial postoperative period was uneventful, with no signs or symptoms of infection, pain, or swelling (Figure 6). Oral hygiene instructions were given again to the patient since some food debris was observed in the splinting area. The sutures on the third molar site were removed, and periapical radiography was performed.

 Four weeks later, positive tooth vitality was observed, and since the splinting on the buccal side was not correctly attached, the surgeon decided to remove the splint. Six months after the surgical procedure, the clinical and radiographic assessments showed an excellent outcome, with positive vitality and no significant mobility of the transplanted tooth.

 The subsequent postoperative appointments showed no signs of inflammation, mobility, or periodontal pathology (probing depths of 3 mm on all sites).

**Figure 7:**
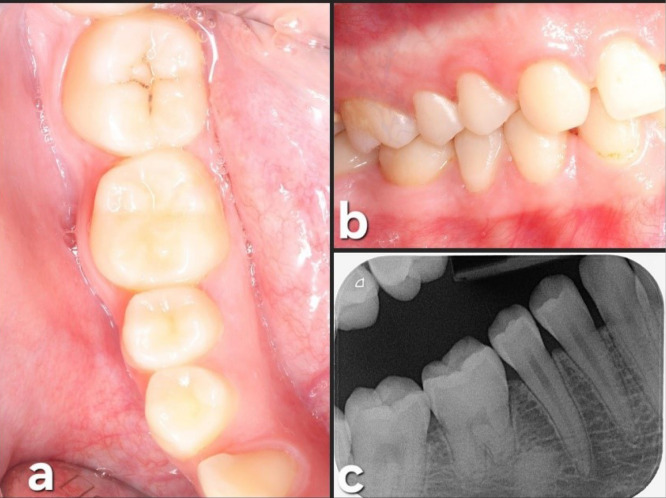
42-month (3.5 years) follow-up. (a) Occlusal view. (b) Lateral view. (c) Periapical radiograph at 42 months showing complete root formation with a complete periodontal ligament (PDL) around the tooth

 In the last follow-up visit (42 months), the autotransplanted tooth exhibited good health, with a normal periodontal and radiographic exploration. Complete root formation was observed (Figure 7).

## Discussion

 This case report introduced a 15-year-old patient with a lower right molar with an incomplete endodontic treatment needing extraction due to the COVID-19 pandemic. The use of 3D imaging and digital tools allowed a more straightforward and predictable treatment. Indeed, the possibility of assessing the shape and size of the third molar enabled the surgeon to predict if the socket would require changes.

 Several therapeutic alternatives are available to replace molars not candidates for restorative procedures or root canal therapy.^16^ Dental implants are one of the most used approaches to replace missing teeth. However, DAT, consisting of placing one autologous tooth in another site within the same individual, may be considered. Indeed, DAT seems to be especially indicated in situations where dental implants are contraindicated. In this regard, the patient’s age is a crucial factor to consider. Furthermore, several reports show that these younger patients seem to have higher success rates when submitted to DAT.^3,4,6^

 This paper underlines the importance of using digital tools combined with 3D imaging through CBCT. The step-by-step process, including isolating donor and receptor teeth, creating 3D models, and conducting measurements, is crucial to increase the predictability and reduce the surgical difficulty when performing DAT.^13^

 Several factors must be considered to ensure the long-term success of these procedures. These include careful case selection, thorough preoperative planning, sufficient bone support and keratinized tissue at the recipient site, and an adequate periodontal state. Additionally, less traumatic surgical techniques and proper oral hygiene can also significantly impact the success of DAT.^9^

 Additionally, factors like the root development stage, the root morphology, the surgery time (especially the time elapsed from the extraction until the final placement of the transplanted tooth), the periodontal status, and the socket shape are important and might influence the success of DAT. Therefore, all these factors should be considered before deciding on the best course of action.^13^

 The detailed description of the surgical procedure, from informed consent to postoperative care, aligns with established protocols outlined in various studies.^13^ The integration of computer-assisted planning is evident in the precision of crown and root sectioning, donor tooth removal, and socket preparation. This echoes findings in studies emphasizing the importance of precise surgical techniques for long-term success.^2^ The emphasis on postoperative care instructions, including a bland diet, ice packs, and medication, aligns with recommendations for optimal recovery observed in similar cases.^13^

 The follow-up section provides valuable insights into the patient’s postoperative journey, with medication adherence, absence of infection signs, and radiographic assessments contributing to validating the success and stability of the DAT procedure. This aligns with findings in long-term follow-up studies emphasizing the significance of regular monitoring and positive clinical outcomes.^13^After extensive research, it is apparent that DAT is a highly dependable and trustworthy treatment method that can result in a favorable long-term prognosis.^4^ Despite its success rate, it is underutilized by many dentists as a regular treatment, but we are confident that further studies can help increase its acceptance.^12^

 One significant advantage of DAT is its suitability for younger patients, as it can align with neighboring teeth during facial growth.^12^ Not only can this method maintain proprioceptive function and a normal periodontal ligament, but it can also produce aesthetic results comparable to other treatments.^10^ Furthermore, it is a cost-effective option that can precede dental implants in bone induction.

 This case report integrates contemporary approaches, including advanced technology and meticulous surgical planning, supported by insights from established studies in DAT. 3D technology has additional benefits, such as an immediate good fit and a significant reduction in recipient zone preparation. Finally, it has resulted in high success and survival rates compared to the conventional method we performed in this new approach for our case report.^17,18^

 It is important to acknowledge that not all patients are eligible for DAT due to the lack of suitable teeth. Consequently, researchers have been exploring alternative approaches, such as using tooth buds as a foundation for new tooth regeneration. Studies have shown that harvesting tooth buds provides a promising platform for developing new teeth, which can be influenced by the odontogenic potential of bone marrow mesenchymal cells. These cells can differentiate into various cell types that form teeth, making them a powerful tool for dental regeneration. The promising results of these studies suggest that the future of dental health is bright, and it is only a matter of time before these innovative methods become widely accepted.^12,13,19^

 Several factors may influence the outcome of surgery. Third molar donor teeth with open apices have shown higher success and survival rates than those with closed apices, as evidenced by our case report using a developing third molar with open apices.^14^ Failures were more common in maxillary sites than in the mandible in the same patient, but the reason remains unclear.^4^

 Various intraoperative factors have been discussed in the literature, such as using bone grafts, donor tooth replicas, the type and duration of splinting protocols, postoperative antibiotics, and the timing of endodontic treatment.^4,5,20,21^ The use of bone graft material has been associated with a higher relative risk of postoperative extraction, which we believe is not advisable for these cases.^4^Studies suggest that using wire composite splinting for more than 14 days reduces the risk of failure and is better suited for donor teeth with complete root formation.^5^ Patients who did not receive antibiotics had a higher failure rate than those receiving antibiotics pre- or postoperatively.^5^ In our case, we prescribed antibiotics one hour before the DAT surgery.

 Postoperative factors such as tooth ankylosis have shown a higher tendency for tooth loss. Fortunately, this complication did not occur in our case.^22^

 For the legal aspect, all these surgeries should be performed with previous detailed informed consent, and if the patient is a minor, their legal representative or tutor should fill out and sign it.

 A randomized controlled trial study with long-term follow-up is essential to achieve more conclusive and detailed results. Also, incorporating photobiomodulation could play a key role in future studies to evaluate the healing process.

## Conclusion

 Despite the limitations of this case report, DAT seems to be a valid option for young patients requiring the extraction of a mandibular molar. The integration of computer-assisted analysis looks extremely useful, reducing the risk of complications, facilitating the surgical procedure and improving the treatment outcomes.

## Competing interests

 The authors did not disclose any conflicts of interest.

## Data Availability Statement

 All data generated or analyzed during this study are included in the published article and/or its supplementary files.

## Ethical Approval

 Ethical approval is not required for individual case reports according to the policies of our institution/journal. Informed consent was obtained from the patient and her parents for publication of this case report and any accompanying images.

## Consent for publication

 Informed consent was obtained from the patient and her parents, including permission to collect and use data for educational purposes such as journal publications, case presentations at educational institutions, medical and dental congresses, intra-oral photographs, radiographic images, and follow-up records. The patient and her parents agreed to all the stated uses, with the only condition being that the patient’s face (extra-oral photographs) and full name would not be disclosed. All authors agreed and consented to publish this case report.
